# Synergistic Effects of a *Tomato chlorosis virus* and *Tomato yellow leaf curl virus* Mixed Infection on Host Tomato Plants and the Whitefly Vector

**DOI:** 10.3389/fpls.2021.672400

**Published:** 2021-05-31

**Authors:** Jie Li, Ji-cheng Wang, Tian-bo Ding, Dong Chu

**Affiliations:** Key Laboratory of Integrated Crop Pest Management of Shandong Province, College of Plant Health and Medicine, Qingdao Agricultural University, Qingdao, China

**Keywords:** *Tomato chlorosis virus*, *Tomato yellow leaf curl virus*, mixed infections, synergism, *Bemisia tabaci* MED

## Abstract

In China, *Tomato chlorosis virus* (ToCV) and *Tomato yellow leaf curl virus* (TYLCV) are widely present in tomato plants. The epidemiology of these viruses is intimately associated with their vector, the whitefly (*Bemisia tabaci* MED). However, how a ToCV+TYLCV mixed infection affects viral acquisition by their vector remains unknown. In this study, we examined the growth parameters of tomato seedlings, including disease symptoms and the heights and weights of non-infected, singly infected and mixed infected tomato plants. Additionally, the spatio-temporal dynamics of the viruses in tomato plants, and the viral acquisition and transmission by *B. tabaci* MED, were determined. The results demonstrated that: (i) ToCV+TYLCV mixed infections induced tomato disease synergism, resulting in a high disease severity index and decreased stem heights and weights; (ii) as the disease progressed, TYLCV accumulated more in upper leaves of TYLCV-infected tomato plants than in lower leaves, whereas ToCV accumulated less in upper leaves of ToCV-infected tomato plants than in lower leaves; (iii) viral accumulation in ToCV+TYLCV mixed infected plants was greater than in singly infected plants; and (iv) *B. tabaci* MED appeared to have a greater TYLCV, but a lower ToCV, acquisition rate from mixed infected plants compared with singly infected plants. However, mixed infections did not affect transmission by whiteflies. Thus, ToCV+TYLCV mixed infections may induce synergistic disease effects in tomato plants.

## Introduction

Tomato (*Solanum lycopersicum* Mill.) is an economically important and widely cultivated crop worldwide. Diseases caused by whitefly-transmitted plant viruses have become a devastating factor in tomato production. The diseases caused by *Tomato yellow leaf curl virus* (TYLCV) and *Tomato chlorosis virus* (ToCV) are particularly noteworthy (Mabvakure et al., 2016; Fiallo-Olivé and Navas-Castillo, 2019). A single-stranded DNA *Begomovirus*, TYLCV seriously impacts tomato production throughout tropical and subtropical regions (Fauquet et al., 2005; Mabvakure et al., 2016). It was first described in 1964 (Cohen and Harpaz, 1964) and has since rapidly spread into new regions near the Indian and Pacific oceans, including Australia, New Caledonia, and Mauritius (Lefeuvre et al., 2010; Mabvakure et al., 2016). A single-stranded RNA *Crinivirus*, ToCV was originally identified in the 1990s in Florida, United States. Subsequently, the virus has spread into more than 20 countries, including those in Europe, Africa, North America, South America, and Asia (Wisler et al., 1998; Bese et al., 2011; Fiallo-Olivé et al., 2011; Navas-Castillo et al., 2011; Arruabarrena et al., 2014; Li et al., 2018; Favara et al., 2019). ToCV and TYLCV can be efficiently transmitted in a semi-persistent and persistent circulative manner by the same whitefly, *Bemisia tabaci* (Gennadius) MED, under natural conditions (Wintermantel and Wisler, 2006; Polston et al., 2014), and the continuous influxes of viruliferous whiteflies is a major factor responsible for the spread of these two viruses (Orfanidou et al., 2016; Shi et al., 2018; Macedo et al., 2019).

The occurrence of ToCV+TYLCV mixed infections has been on the rise in recent years, and ToCV+TYLCV mixed infected tomatoes were reported as early as 2007 in Cuba (Martíne-Zzubiaur et al., 2008). At present, the co-occurrence of ToCV and TYLCV in tomatoes has become widespread, as determined by field observations, in many Chinese provinces, including Shandong, Tianjin, Hebei, Jiangsu, and Yunnan (Dai et al., 2017; Liu et al., 2018). The occurrence of ToCV+TYLCV mixed infections in China has been closely associated with their vector, the whitefly *B. tabaci* MED, which is the main whitefly species in China. It greatly promotes the spread of these two viruses in the field (Wei et al., 2019). However, little information is currently available regarding the effects of ToCV+TYLCV mixed infections on whitefly-mediated transmission and host plant growth.

Multiple viral infections of the same host plant are ubiquitous in the field and frequently result in synergism or antagonism among the different viruses – usually with unpredictable pathological consequences (DaPalma et al., 2010). Multiple viral infections may lead to changes in the cumulative titers in one or more of the involved viruses, cause a variety of diverse symptoms in the host plant or, in the worst cases, instigate outbreaks of new epidemics (e.g., Pruss et al., 1997; Wintermantel, 2005; Garciìa-cano et al., 2006; Wintermantel et al., 2008; Syller, 2012; Li et al., 2014; Mascia and Gallitelli, 2016; Favara et al., 2019). For example, a mixed ToCV and *Tomato infectious chlorosis virus* (TICV) infection in *Nicotiana benthamiana* results in plant death, an increased TICV titer and decreased ToCV titer relative to single infections (Wintermantel et al., 2008). In other cases, certain plant viruses only destroy hosts when they act in cooperation with other independent plant viruses (Abdullah et al., 2017). For example, obligate mutualism between a potato virus and *Tobacco mosaic virus* causes a high mortality rate and defoliation streak in young tomato leaves (Hull, 2014). Furthermore, multiple viral infections enhance plant virus persistence by supporting greater viral reproduction rates, which increases the chance of the host becoming an inoculation source in the next growing season. In such a case, a mixed infection of two *African cassava mosaic virus* strains in *N. benthamiana* plants results in symptoms covering all of the leaves, whereas single-strain infections exhibit only partial coverage, with some leaves remaining totally asymptomatic (Fondong et al., 2000). However, the mechanisms of different mixed viral infections are poorly understood.

The simultaneous presence of large whitefly populations in the tomato fields of many tropical and subtropical regions worldwide and their begomoviruses and criniviruses transmission efficiencies suggest that ToCV+TYLCV mixed infections can occur where these viruses are present. However, to date, little is known regarding the synergistic interactions among different viruses in tomato, or on how mixed viral infections affect plant physiology and vector viral acquisition. The aims of this report were to study the synergistic interactions between the single-stranded DNA virus TYLCV and single-stranded RNA virus ToCV in tomato, the spatio-temporal dynamics of the viruses in tomato plants and the effects of mixed viral infections on vector viral acquisition and transmission.

## Materials and Methods

### Plants, Viruses, and Insects

Tomato seeds (*S. lycopersicum* Mill. cv. Zhong Za 9) were purchased from the Chinese Academy of Agricultural Sciences, Beijing, China. Cotton seeds (*Gossypium hirsutum* M. cv. Lu-Mian 28) were purchased from Shandong Luxi Technology Co., Ltd., Shandong, China. All the plants were cultivated in a greenhouse under a 16-h:8-h light:dark photoperiod at 27 ± 2°C. Tomato plants that had reached the 4–5 true-leaf stage and cotton plants reaching the 2–3 true-leaf stage were used in the experiments. All the plants were watered every 5–7 days as necessary.

The ToCV and TYLCV isolates were collected from infected field-grown tomatoes in Qingdao, Shandong Province, China, transmitted by *B. tabaci* MED and maintained on “Zhong Za 9” tomato plants grown in climate-controlled cubicles (27 ± 2°C; 16-h:8-h light:dark photoperiod) in the laboratory.

The *B. tabaci* MED population was obtained from a laboratory colony established from prior field collections. The population was reared on cotton plants (*G. hirsutum* M. cv. Lu-Mian 28) at 27 ± 2°C under a 16-h: 8-h light:dark photoperiod. The purity of the population was monitored every 30 days using the *Vsp* I-based mtCOI PCR–RFLP method (Chu et al., 2012).

### Viral Inoculation

The independently ToCV- and TYLCV-infected tomato plants were obtained by inoculating non-infected tomato plants with 20 viruliferous male whiteflies that had all emerged within a 24-h period and were allowed to feed for 48-h on ToCV- and TYLCV-infected tomato plants, respectively. Whiteflies were transferred to tomato plants using a clip-on cage placed on the first true leaf and there was a 10-day inoculation access period. Using the same method, ToCV+TYLCV mixed infected tomato plants were obtained by inoculating control (non-infected) tomato plants simultaneously with 20 ToCV- and 20 TYLCV-viruliferous male whiteflies. Similarly, non-infected tomato plants (mock treatment) were obtained by allowing 20 non-viruliferous male whiteflies to feed on each plant for a comparable period of time. After inoculation, plants were maintained in a greenhouse at 27 ± 2°C under a 16-h: 8-h light:dark photoperiod. Plants from each of four treatment groups (non-infected, ToCV-infected, TYLCV-infected, and ToCV+TYLCV mixed infected tomato plants) were used in all the experiments.

### Observation of Plant Growth Characteristics and Measurement of the Disease Severity Index

At 42 days after inoculation, the plant growth characteristics, including the plant heights and total fresh weights of aboveground tissues, were evaluated in each treatment group. Plant height was determined as a measure of stem length (cm) from soil level to stem tip (Murphy and Bowen, 2006). Plant total fresh weight of aboveground tissues was determined by cutting the stem at the soil line and immediately weighing all associated tissues. For each of the four treatments, there were three trials, with eight plants per trial.

The disease severity index (DSI) was assessed at 6, 9, 12, 15, 18, 21, 24, 27, 30, 33, 36, 39, and 42 days post-infection (dpi) by *B. tabaci* MED carrying the pathogen. A modified 0–5 arbitrary rating scale was used, as described by Duc and Posta (2018), as follows: 0, 1, 2, 3, 4, 5, and 6 showed no (0%), ≤10%, 11–25%, 26–49%, 50–74%, 75–99%, and all (100%) leaves, respectively, having yellowing and crimping. The DSI was calculated from the disease severity ratings of 30 plants (three replicates of 10 plants per treatment) (Thompson et al., 2011) of the inoculated plants using the following formula (Raupach, 1996):

$$\mathrm{DSI}\left(\%\right)=\sum \left\{\frac{\left(P\times Q\right)}{\left(M\times N\right)}\times 100\right\}$$

where *P* represents the rating number, *Q* represents the number of plants having a rating, *M* represents the total number of plants and *N* represents the highest rating.

### Primer Design and Plasmid Standard Preparations for Real-Time PCR (qPCR)

Highly conserved regions of TYLCV (GenBank no. KM435327.1) and ToCV (GenBank no. KC709510.1) were selected for the absolute quantification of TYLCV and ToCV, respectively, using the qPCR design tools from Integrated DNA Technologies Inc. (Coralville, IA, United States). The sequence and nucleotide coordinates of primers are provided in Supplementary Table 1. To obtain the insertion fragment for ToCV plasmid standards, previously obtained ToCV cDNA was amplified using the primer pair ToCV-qS3/ToCV-qA3, which produces a fragment of 157 bp, The PCR conditions were as follows: 95°C for 4 min, followed by 35 cycles of 95°C for 30 s, 57°C for 30 s, and 72°C for 20 s, with a final extension at 72°C for 7 min. After purifying the amplified fragments, they were cloned into the pMD^TM^ 18-T vector (Takara Biotechnology Dalian Co., Ltd., China) and transformed into *Escherichia coli* DH5α competent cells. The PCR product’s insertion was verified by PCR screening and sequencing. Then, plasmid extractions were performed using a TIANpure Mini Plasmid Kit (Tiangen Biotech Beijing Co., Ltd.). The purity and concentration were measured using a Nano Photometer N60 (Implen Scientific Inc., Germany). A dilution series of 3.15 × 10^8^ to 3.15 × 10^3^ copies per μL was made for standard samples to develop a standard curve for the absolute quantification of ToCV genomic mRNA copies. The molecular copies of the standard samples were calculated using the following the formula (Shirima et al., 2017):

$$\mathrm{Plasmid}\,\mathrm{copy}\,\mathrm{number}/\mu \,L=\frac{6.02\times 10^{23}\times \mathrm{plasmid}\,\mathrm{amount}\,\left(\mathrm{ng}/\mu \,L\right)}{\mathrm{NW}\times 10^{9}}$$

where *NW* represents the plasmid molecular weight expressed as plasmid size (bp) × molar mass per base (650 g mol^–1^ bp^–1^). The plasmid amount was calculated from the plasmid concentration determined using a Nano Photometer N60.

The construction of the recombinant TYLCV plasmid for standards was accomplished with the same procedure used for ToCV. The insertion fragment for TYLCV was amplified using the primer pair TYLCV-F/TYLCV-R, which produced a fragment of 194 bp. The PCR conditions were as follows: 95°C for 4 min, followed by 35 cycles of 95°C for 30 s, 57°C for 30 s, and 72°C for 30 s, with a final extension at 72°C for 7 min. A dilution series from 2.56 × 10^8^ to 2.56 × 10^3^ copies per μL of TYLCV plasmid was made for standard samples to develop a standard curve for the absolute quantification of TYLCV genomic DNA copies.

### Viral DNA Extraction and TYLCV Quantification Using qPCR

*Tomato yellow leaf curl virus* was quantified by qPCR in accordance with the method used by Wang et al. (2014). TYLCV-infected and ToCV+TYLCV mixed infected tomato plant upper (the second leaf from the top) and lower (the second leaf from the bottom) leaves were collected at 14 and 28 dpi and stored in liquid nitrogen, and total DNA was extracted using the TIANamp Genomic DNA Kit in accordance with the manufacturer’s protocol (Tiangen Biotech Beijing Co., Ltd., China). The DNA concentration was measured using a NanoPhotometer N60. The TYLCV genomic DNA’s abundance was determined using qPCR with the primers TYLCV-F/TYLCV-R. qPCR was performed on a qTOWER 2.0 Real-time PCR Thermal Cycler (Analytik Jena AG, Germany) with SYBR green detection (Takara Biotechnology Dalian Co., Ltd., China). The qPCR mixture consistent of 10 μL SYBR^®^ Premix Ex Taq, 0.4 μL each primer TYLCV-F/TYLCV-R (10 μM) (Supplementary Table 1), 2 μL genome DNA (2.5 ng/μL) and sufficient RNase-free ddH_2_O to yield a final volume of 20 μL. Thermal cycling conditions were as follows: 95°C for 2 min, followed by 40 cycles of 95°C for 15 s and 60°C for 30 s. All amplicons had only a single dissociation peak. There were four treatments, each with three trials and eight plants per trial. Each gene was analyzed using two technical replicates per sample. The cycle threshold and copy number were determined using Analytik Jena’s qPCRsoft 3.2 software (Analytik Jena AG, Germany). Amplification was followed by a melt-curve analysis. The average TYLCV accumulation (copy number per μL) was calculated per sample in accordance with the absolute quantity standard curve (*Y* = −3.53X + 38.58; slope = −3.53, intercept = 38.58, amplification efficiency = 0.92 and *R*^2^ = 0.997).

### Viral RNA Extraction and ToCV Quantification by Reverse Transcription qPCR (RT-qPCR)

Total RNA was extracted from ToCV-infected and ToCV+TYLCV mixed infected tomato plant upper (the second leaf from the top) and lower (the second leaf from the bottom) leaves at 14 and 28 dpi using TRIzol (Invitrogen, Boston) in accordance with the manufacturer’s instructions. The RNA concentration was confirmed using a NanoPhotometer N60. cDNA was synthesized from the total RNA using a PrimeScript^TM^ RT reagent Kit with gDNA Eraser in accordance with the manufacturer’s protocol (Takara Biotechnology Dalian Co., Ltd., China). The RT reaction mixture consistent of 2 μg total RNA and DEPC sterile water was added to form a final 1/10 cDNA dilution. cDNA was then stored at −20°C until used. The RT-qPCR was conducted on a qTOWER 2.0 Real-time PCR Thermal Cycler using SYBR^®^ Premix Ex TaqTM (Tli RNaseH Plus) detection (Takara Biotechnology Dalian Co., Ltd., China). The ToCV mRNA abundance was determined using RT-qPCR with the primers ToCV-qS3/ToCV-qA3 (Supplementary Table 1). The RT-qPCR mixture consisted of 10 μL SYBR^®^ Premix Ex Taq, 0.4 μL each ToCV-qS3/ToCV-qA3 primers (10 μM), 2 μL cDNA and sufficient RNase-free ddH_2_O to yield a final volume of 20 μL. Thermal cycling conditions were as follows: 95°C for 2 min, followed by 40 cycles of 95°C for 15 s and 60°C for 30 s. All the amplicons had only a single dissociation peak. Each gene was analyzed using two technical replicates per sample. The cycle threshold and copy number were determined using Analytik Jena’s qPCRsoft 3.2 software. Amplification was followed by a melt-curve analysis. The average ToCV accumulation (copy number per μL) per sample was calculated in accordance with the absolute quantity standard curve (*Y* = −3.4X + 37.14; slope = −3.4, intercept = 37.14, amplification efficiency = 0.97 and *R*^2^ = 0.996).

### Efficiencies of TYLCV and ToCV Acquisition by Whiteflies

Tomato plants at the 7–8 true-leaf stage that had been infected witheither TYLCV or ToCV for 14 days were used for this procedure. Newly emerged (0–24 h) non-viruliferous female adultwhiteflies were first placed in small self-sealing Petri dishes (10-cm diameter) containing moistened filter paper for the 3-h preacquisition starvation period. They were then moved onto TYLCV-infected, ToCV-infected or ToCV+TYLCV mixed infected tomato plants for a 6-, 12-, 24-, or 48-h timed acquisition feeding periods. Each viral acquisition test was conducted using 60 adult whiteflies. When the acquisition efficiency of TYLCV by whiteflies was tested, the whiteflies were placed on TYLCV-infected and ToCV+TYLCV mixed infected tomato plant upper leaves (the second leaf from the top). When the acquisition efficiency of ToCV by whiteflies was tested, the whiteflies were placed on lower leaves(the second leaf from the bottom) of ToCV infected and ToCV+TYLCV mixed infected tomato plants. Thereafter the adults were collected, stored at −70°C and later assayed individually for detectable TYLCV or ToCV using PCR methods with primers specific for ToCV (ToCV-F1/ToCV-R1) or TYLCV (TYLCV-F1/TYLCV-R1) (Supplementary Table 1). Finally, viral acquisition rates (no. infected/no. inoculated) from singly or doubly infected tomato plants were calculated.

### Viral Transmission by *Bemisia tabaci* MED

*Tomato chlorosis virus*-infected, TYLCV-infected and ToCV+TYLCV mixed infected tomato plants were used as viral sources at 4 weeks post-inoculation (wpi). All the tomato plants were infected in the 4 true-leaf stage. Large vector whitefly populations were allowed to mass feed on virus-infected source plants for 24-h acquisition access periods. Then, clip-on cages containing 20 female adults each were used for transmission experiments by attaching each cage to the underside of leaf for 48-h. Following inoculation, cages were removed from leaves, and the remaining whiteflies were killed. Then, the leaf on which the whiteflies fed was removed at 1 wpi to prevent the possible maturation of whitefly nymphs. At 4 wpi, plants were analyzed for the presence of ToCV and TYLCV as described previously (TYLCV quantification by qPCR and ToCV quantification by reverse transcription qPCR).

### Statistical Analyses

Statistical analyses of plant DSIs, as well as heights and fresh weights of virus-infected plants, for significance were accomplished by one-way ANOVAs using Tukey’s honestly significant difference (HSD) tests at a 0.05 level. The viral accumulations in tomato plants at the same stage were compared using *t*-tests with a Bonferroni correction. Viral acquisition and transmission rates by the vector were analyzed using Pearson’s chi-square test. All the data analyses were conducted using the software SPSS 16.0 (IBM, Armonk, NY, United States).

## Results

### The DSIs and Symptoms in ToCV and TYLCV Mixed Infected Tomato Plants

**FIGURE 1:**
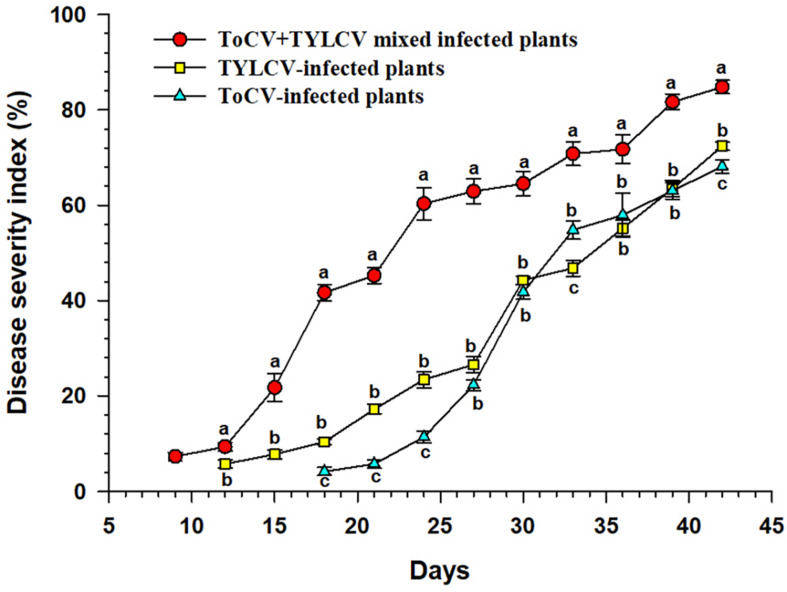
Disease severity index of tomato plants at different times after infection with different viruses. Letters on means at the same day, when different, indicate a significant difference according to one way ANOVA at *P* < 0.05.

The ToCV+TYLCV mixed infected plants began to show symptoms at 9 dpi, whereas the TYLCV-and ToCV-infected plants began to show symptoms at 12 and 18 dpi, respectively (Figure 1). The DSI of the ToCV+TYLCV mixed infected plants was significantly greater than those of singly TYLCV- and ToCV-infected plants at each time post-infection (*P* < 0.05) (Figure 1). The DSI of the TYLCV-infected plants was significantly greater than that of the ToCV-infected plants at 18, 21, 24, 33, and 42 dpi (*P* < 0.05), but there were no significant differences in DSIs at 27, 30, 36, and 39 dpi (*P* > 0.05) (Figure 1).

**FIGURE 2:**
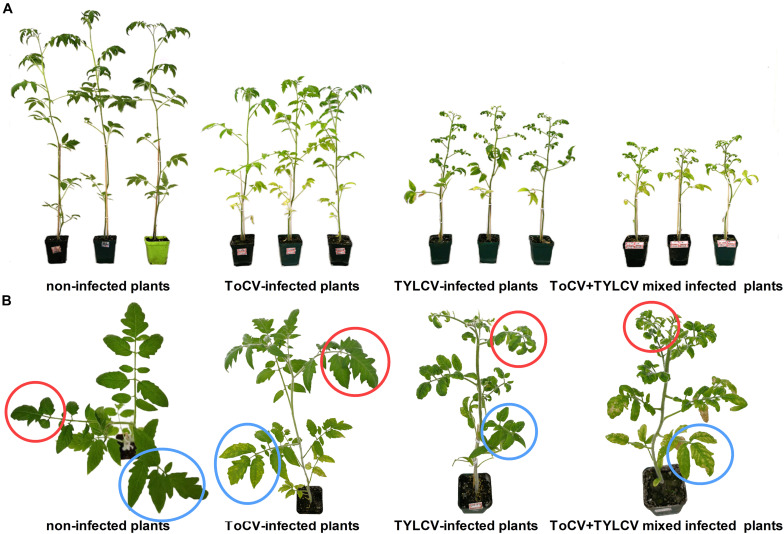
Symptoms of non-infected and virus-infected tomato plants after 42 days post-inoculation. **(A)** Side view of tomato plants. **(B)** Zoom of the top view of tomato plants. Red circles, upper leaves; blue circles, lower leaves.

At 42 dpi, visual observations of plants revealed clear differences in growth between singly infected, with ToCV or TYLCV, tomato plants and plants infected with both ToCV and TYLCV (Figure 2). Non-infected tomato plants exhibited the best overall growth, with no wilting or yellowing of their leaves. In ToCV-infected tomato plants, the primary symptom was the chlorotic lower leaves that became desiccated and then died. In TYLCV-infected tomato plants, the main symptom was chlorotic and deformed upper leaves. Tomato plants infected with both ToCV and TYLCV exhibited a combination of symptoms, with chlorotic and deformed upper leaves and chlorotic lower leaves that became desiccated and then died (Figure 2).

### Synergism Demonstrated by Plant Height and Fresh Weight

**FIGURE 3:**
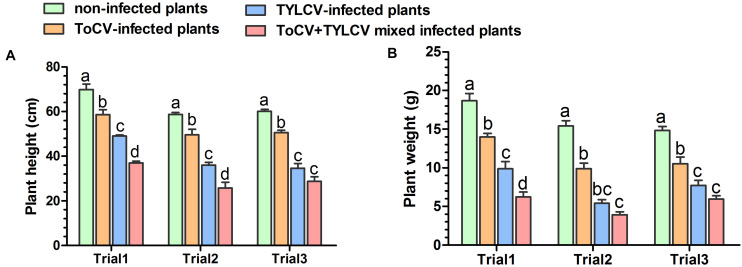
Heights and aboveground fresh weights of non-infected and virus-infected tomato plants after 42 days post-inoculation. **(A)** Plant heights. **(B)** Plant weights. Letters on means in the same trial, when different, indicate a significant difference according to one way ANOVA at *P* < 0.05.

Stem heights and aboveground fresh weights of plants in each treatment were compared at 42 dpi. In each of the three trials, the stem heights and final aboveground fresh weights of ToCV+TYLCV mixed infected plants were significantly less than those of plants infected with ToCV alone (*P* < 0.05) (Figure 3). In Trial 3, there was no significant difference in stem heights and final aboveground fresh weights between ToCV+TYLCV mixed infected and TYLCV-infected plants (*P* > 0.05) (Figure 3). In addition, plants infected with either virus alone or with ToCV+TYLCV resulted in lower stem heights and aboveground fresh weights than non-infected controls (*P* < 0.05) (Figure 3).

### Absolute Quantification of TYLCV and/or ToCV in Single and Mixed Infections of Tomato Plants

**FIGURE 4:**
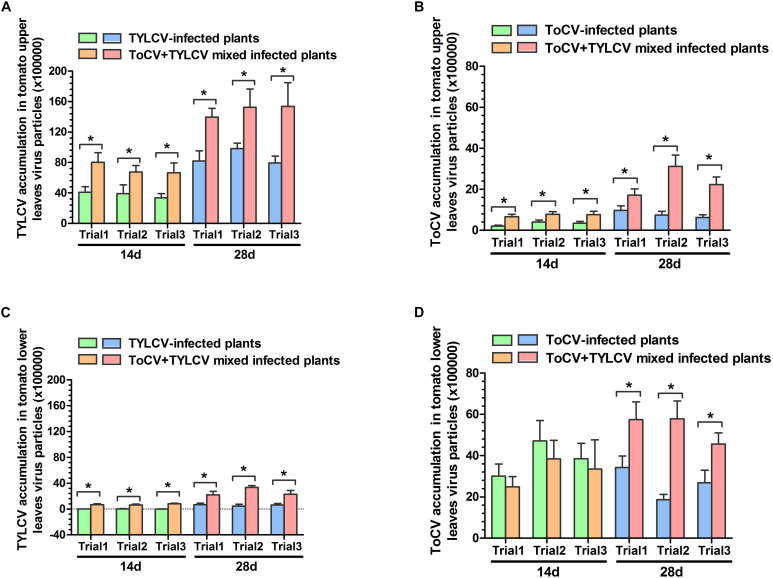
*Tomato chlorosis virus* (ToCV) and *Tomato yellow leaf curl virus* (TYLCV) accumulations in different virus-infected tomato plants. **(A)** ToCV accumulation in upper leaves of ToCV-infected and ToCV+TYLCV mixed infected plants. **(B)** ToCV accumulation in lower leaves of ToCV-infected and ToCV+TYLCV mixed infected plants. **(C)** TYLCV accumulation in upper leaves of TYLCV-infected and ToCV+TYLCV mixed infected plants. **(D)** TYLCV accumulation in lower leaves of TYLCV-infected and ToCV+TYLCV mixed infected plants. Asterisks indicate a significant difference between single infection and mixed infection (Student’s *t*-test, *P* < 0.05).

In each of the three trials, TYLCV accumulations in upper and lower leaves of ToCV+TYLCV mixed infected tomato plants were significantly greater than those in TYLCV singly infected tomato plants (*P* < 0.05) (Figures 4A,C). Similarly, ToCV accumulations in upper leaves of ToCV+TYLCV mixed infected tomato plants were significantly greater than those in ToCV singly infected tomato plants (*P* < 0.05) (Figure 4B). In lower leaves, ToCV accumulations in ToCV+TYLCV mixed infected plants were significantly greater than those in ToCV-infected plants at 28 dpi in each of the three trials (Figure 4D). In addition, there were greater TYLCV accumulations in the upper leaves of tomato plants harboring single and mixed infections than in lower leaves at the same post-infection day in each trial (Figures 4A,C). In contrast, there were lower ToCV accumulations in the upper leaves of tomato plants harboring single and mixed infections than in lower leaves at the same post-infection day in each trial (Figures 4B,D). Furthermore, TYLCV and ToCV accumulations in both upper and lower leaves rose continuously from 14 to 28 dpi in single and mixed infections (Figure 4), except ToCV accumulations at 14 dpi in the lower leaves of ToCV-infected and ToCV+TYLCV mixed infected plants (Figure 4D).

### Viral Acquisition Rate of the Whitefly Vector

**FIGURE 5:**
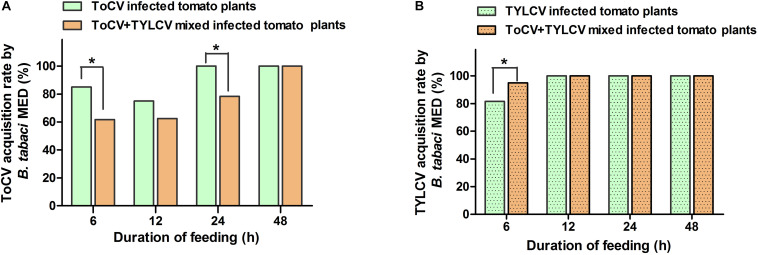
Virus acquisition rates of *Bemisia tabaci* feeding on virus infected tomato plants at different time. **(A)** *Tomato chlorosis virus* (ToCV) acquisition rate of *B. tabaci* feeding on ToCV-infected and ToCV+TYLCV mixed infected plants. **(B)** *Tomato yellow leaf curl virus* (TYLCV) acquisition rate of *B. tabaci* feeding on TYLCV-infected and ToCV+TYLCV mixed infected plants. Asterisks indicate a significant difference between single infection and mixed infection (chi square test, *P* < 0.05).

After a 6-h access exposure period to infected leaves, the acquisition rate of TYLCV by whitefly on ToCV+TYLCV mixed infected tomato plants was significantly greater than on plants infected with only TYLCV (*X*^2^ = 4.876, df = 1, *P* < 0.05) (Figure 5B). In contrast, the acquisition rate of ToCV by whitefly on ToCV+TYLCV mixed infected tomato plants was significantly lower than on ToCV-infected plants (*X*^2^ = 8.352, df = 1, *P* < 0.05) after 6- and 24-h access exposure periods (Figure 5A). The acquisition rate of TYLCV by *B. tabaci* MED increased with the length of the access period on both TYLCV-infected and ToCV+TYLCV mixed infected tomato plants and reached 100% after a 12-h access period (Figure 5B). The acquisition rate of ToCV by *B. tabaci* MED increased with the length of the access period and reached 100% after a 24-h access period on ToCV-infected tomato plants, but required 48-h to reach 100% on ToCV+TYLCV mixed infected tomato plants (Figure 5A).

### Transmission Efficiencies of ToCV and TYLCV by *B. tabaci* MED

**FIGURE 6:**
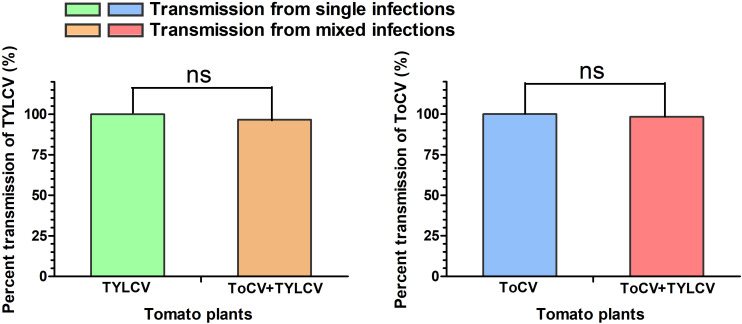
Percent transmission of *Tomato chlorosis virus* (ToCV) and *Tomato yellow leaf curl virus* (TYLCV) by *Bemisia tabaci*. ns indicate no significant difference between single infection and mixed infection (chi square test, *P* < 0.05).

In transmission experiments conducted immediately after a 24-h viral acquisition period on virus-infected tomato plants, ToCV and TYLCV were transmitted with equal efficiencies (100%) from singly infected plants (Figure 6). Similarly, transmission from ToCV+TYLCV mixed infected plants resulted in 59 (98.3%), 58 (96.7%), and 58 (96.7%) of 60 new plants developing ToCV, TYLCV, and ToCV+TYLCV mixed infections, respectively (Figure 6).

## Discussion

The increased incidences of ToCV, TYLCV, and their whitefly vector in greenhouse and field production systems across numerous vegetable crops highlights the need for additional efforts to manage the two viruses (Wu et al., 2016; Liu et al., 2018). Efforts to elucidate the relationships among ToCV, TYLCV, their host plants and their vector *B. tabaci* MED are important for understanding virus epidemiology and developing effective virus-control management strategies.

Here, ToCV and TYLCV accumulations in mixed infected tomato plants were greater than those in ToCV or TYLCV singly infected tomato plants, suggesting that a pattern of synergism between ToCV and TYLCV promotes their replication and these viruses overcome the resistance of host plant. RNA silencing serves as an antiviral defense system in response to viral challenges in plants, and RNA silencing suppressors (RRSs) are crucial factors used by viruses to overcome host silencing and infect host plants (Alvarado and Scholthof, 2009). Viral synergism is often associated with the actions of RSS proteins (Pruss et al., 1997; Vanitharani et al., 2004; Srinivasan and Alvarez, 2007). The crinivirus *Sweet potato chlorotic stunt virus* cause synergistic diseases with other begomoviruses, leading to their increased accumulation levels. This increase is mediated by the *Sweet potato chlorotic stunt virus*-encoded RNase3 protein (functioning as an RSS), which also mediates synergistic diseases with two other unrelated RNA viruses (Cuellar et al., 2009, 2015). Whether RNA silencing occurred as a result of the synergism between ToCV (crinivirus) and TYLCV (begomovirus) in host plants requires further investigation. In addition, the crinivirus ToCV, which can be transmitted in a semipersistent manner, causes synergistic diseases with *Tomato severe rugose virus*, which can be transmitted in a persistent circulative manner, in tomato plants. This shortens the latent periods and prolongs the incubation periods of both viruses, contributing to more rapid viral spread (Favara et al., 2019). In this study, ToCV and TYLCV mixed infection-induced disease symptoms appeared earlier compared with single infections. Thus, the mixed infection may shorten the latent periods of both viruses, although further studies are needed to confirm such an effect.

The mixed infection resulted in increased accumulations of ToCV and TYLCV, induced more severe symptoms, increased the tomato plant DSI (Figure 1) and decreased tomato plant height and fresh weight (Figure 3), demonstrating that the interactions between ToCV and TYLCV in tomato plants are synergistic. Thus, ToCV+TYLCV mixed infections may explain why virus-infected tomato plants in some fields exhibit severe yellowing, curled leaves and reduced fruit production (Hu et al., 2020). Synergistic interactions have also been reported between different viruses (Anjos et al., 1992; Anderson et al., 1996; Scheets, 1998; Stenger et al., 2007; Wintermantel et al., 2008). For example, ToCV and *Tomato spotted wilt virus* (TSWV) mixed infected plants accumulate two to three times less biomass compared with singly or non-inoculated plants (Garciìa-cano et al., 2006). Similarly, mixed infections of *Cucumber mosaic virus* and *Pepper mottle virus* reduce the stem heights, aboveground fresh weights and yields of bell pepper plants relative to each virus alone (Murphy and Bowen, 2006). Moreover, each viral treatment results in a characteristic systemic symptom, and the mixed infections are extremely severe but not lethal. Here, the symptoms of ToCV+TYLCV mixed infections appeared earlier than those of single infections and that two kinds of viral symptoms, curling upper and chlorotic lower leaves curling, accompanied by plant, were produced. These conditions were not evident in single infections. This is similar to the report by Fondong et al. (2000) in which mixed infections of the *East African cassava mosaic virus* and *African cassava mosaic virus* increased the viral disease symptoms in the host plants. However, our results differ from previous studies in which mixed infections with ToCV and either TSWV or TICV resulted in crop failure, but new symptoms were not observed (Garciìa-cano et al., 2006; Wintermantel et al., 2008). The increased severity of symptoms in mixed infected tomato plants may affect the preference of virus vectors. We found that whitefly preferred ToCV+TYLCV mixed infected tomato plants to non-infected and singly infected tomato plants (unpublished results). This is consistent with a previous report. Additionally, symptoms caused by different mixed viral infections may be affected by environmental conditions, such as climatic variations, insecticide spraying and insect vectors (Tatineni et al., 2010; Guo et al., 2017; Macedo et al., 2019).

Our research also indicated that mainly the upper parts of tomato plants showed TYLCV-related symptoms, whereas mainly the lower parts of tomato plant showed ToCV-related symptoms in the early stage of ToCV+TYLCV mixed infections (Figure 2). The results of qPCR also indicated that the accumulation of TYLCV in upper parts of mixed infections was significantly greater than in lower parts, whereas the accumulation of ToCV in upper parts of mixed infections was significantly less than in lower parts (Figure 4). These results showed that the viral symptoms are positively correlated with the viral accumulation, and ToCV and TYLCV occupy different niches in the plant. Therefore, they do not compete with each other, which is more conducive to the replication of both viruses in the plants. This explains part of the synergistic effects observed in the mixed infections from a new perspective.

The effects of synergistic interactions during mixed viral infections on viral acquisition and transmission by the vector deserve special attention, because they may have serious ecological and epidemiological consequences. The increased accumulation of one or both viruses in mixed infections may result in increased vector transmission (Froissart et al., 2010). For example, the transmission efficiencies of ToCV and TICV by whiteflies corresponded to viral accumulation levels in hosts in both single and mixed infections (Wintermantel et al., 2008). In this study, ToCV+TYLCV mixed infections resulted in greater TYLCV, but not ToCV, acquisition rates by whiteflies (Figure 5). However, the mixed infection did not affect the transmission of both viruses by whiteflies (Figure 6). The reason for the high transmission efficiency of viruses by vectors from different virus infected plants may be that small whitefly populations was used for viral transmission in this study. It may also be related to the time of viral acquisition by vectors. Thus, viral accumulation in plants is not the only factor influencing the efficiency of vector acquisition, and the acquisition efficiency may be associated with host plant nutrition and defense responses to insect herbivores. Viral infections elevate plant amino acid and soluble carbohydrate concentrations and subsequently affect herbivorous insect biology (Wilkinson and Douglas, 2003; Xu et al., 2014). In contrast, in some double infections, both or at least one of the viruses may enhance viral accumulation and broaden the virus distribution in host plants, thereby increasing the availability of the virus to feeding vectors (Mascia et al., 2010). Moreover, double viral infections may affect the biology and preferences of virus vectors. For example, *Potato virus Y* (PVY) and *Potato leafroll virus* (PLRV) mixed infected plants increase the fecundity of *Myzus persicae* and *Macrosiphum euphorbiae*, which both preferentially settle on PVY+PLRV mixed infected plants compared with PVY and PLRV singly infected plants (Srinivasan and Alvarez, 2007). Here, *B. tabaci* MED also preferentially settled on ToCV and TYLCV mixed tomato plants compared with singly infected plants, but ToCV and TYLCV mixed tomato plants decreased the fecundity of *B. tabaci* MED (unpublished results). It is probable that the visual and/or olfactory stimuli emitted by mixed infected plants are more attractive to whiteflies than the stimuli emitted by singly infected plants. Plant-mediated interactions between ToCV and TYLCV have far-reaching implications in disease epidemiology, because the two viruses often occur in mixed infections (Liu et al., 2018; Hu et al., 2020).

In this study, ToCV and TYLCV simultaneously infected tomato plants, resulting in synergistic effects. Previous studies have reported that the time and sequence of viral inoculations may affect the relationships among different viruses in host plants. For example, pre-infection with ToCV resulted in the susceptibility of the host cultivar to TSWV, whereas a simultaneous mixed inoculation did not (Garciìa-cano et al., 2006). Whether changes in the sequence of ToCV and TYLCV inoculations alter their synergism is still unclear. Moreover, synergistic interactions that enhance virus pathogenicity may increase plant damage, especially in susceptible cultivars, thereby increasing crop yield losses (Murphy and Bowen, 2006; Malik et al., 2010; Tatineni et al., 2010). In this study, ToCV+TYLCV mixed infected plants induced disease synergism. Although there are a large number of TYLCV-resistant cultivars on the market, the resistance levels to ToCV are not yet known and need to be determined. However, currently, no cultivar has demonstrated resistance to both ToCV and TYLCV. Therefore, breeding resistant cultivars may be an effective method to control this synergistic disease.

## Data Availability Statement

The raw data supporting the conclusions of this article will be made available by the authors, without undue reservation.

## Author Contributions

JL and DC conceived and designed the research. JL, J-CW, and T-BD conducted the experiments. JL wrote the manuscript and analyzed the data. All authors read and approved the manuscript.

## Conflict of Interest

The authors declare that the research was conducted in the absence of any commercial or financial relationships that could be construed as a potential conflict of interest.

## Supplementary Material

Supplementary Table 1 Primers for qPCR of *Tomato chlorosis virus* (ToCV) and *Tomato yellow leaf curl virus* (TYLCV).
