# Supplementary material for: Synergistic Effects of a Tomato chlorosis virus and Tomato yellow leaf curl virus Mixed Infection on Host Tomato Plants and the Whitefly Vector
Source: Front Plant Sci. 2021 May 31;12:672400. doi: 10.3389/fpls.2021.672400 (PMC8201402; doi:10.3389/fpls.2021.672400)
Supplement: Supplementary Table 1 — Primers for qPCR of Tomato chlorosis virus (ToCV) and Tomato yellow leaf curl virus (TYLCV). [file Table_1.DOCX]

**Supplementary materials:**

Table S1. Primers for qPCR of *Tomato chlorosis virus* (ToCV) and *Tomato yellow leaf curl virus* (TYLCV)

| Primer | Nucleotide sequence (5'-3') | Position |
| --- | --- | --- |
| ToCV-qS3 | GGCAGGTTCGTGGACCATAA | 6,110-6,130^a^ |
| ToCV-qA3 | ACTTTCCAATCGCGTCCCAT | 6,247-6,267 |
| TYLCV-F | ATTCGGAAGTGGAGAAAACAT | 1,273-1,294^b^ |
| TYLCV-R | GAACTCATCACTGCTCCTCAG | 1,446-1,467 |
| ToCV-F1 | GGTCAATTATGAGGTCGTGAA | 372-392^a^ |
| ToCV-R1 | CTCTGCCCAGACTTGTAATCA | 822-842 |
| TYLCV-F1 | ACTTCGACAGCCCATACAGC | 963-982^b^ |
| TYLCV-R1 | GAAACCTATCCCGCAAATCA | 1,174-1,193 |
| ^a^ Nucleotide positions correspond to the genomic RNA sequence of ToCV (GenBank accession no. KC709510.1) | | |
| ^b^ Nucleotide positions correspond to the genomic RNA sequence of TYLCV (GenBank accession no. KM435327.1) | | |
